# FPGA-Based Hybrid-Type Implementation of Quantized Neural Networks for Remote Sensing Applications

**DOI:** 10.3390/s19040924

**Published:** 2019-02-22

**Authors:** Xin Wei, Wenchao Liu, Lei Chen, Long Ma, He Chen, Yin Zhuang

**Affiliations:** 1 Beijing Key Laboratory of Embedded Real-time Information Processing Technology, Beijing Institute of Technology, Beijing 100081, China; weixin@bit.edu.cn (X.W.); 2220170351@bit.edu.cn (L.C.); chenhe@bit.edu.cn (H.C.); 2 School of Information Engineering, Zhengzhou University, Zhengzhou 450001, China; ielongma@zzu.edu.cn; 3 School of Electronics Engineering and Computer Science, Peking University, Beijing 100087, China

**Keywords:** remote sensing, convolutional neural network, hybrid-type inference, symmetric quantization, FPGA

## Abstract

Recently, extensive convolutional neural network (CNN)-based methods have been used in remote sensing applications, such as object detection and classification, and have achieved significant improvements in performance. Furthermore, there are a lot of hardware implementation demands for remote sensing real-time processing applications. However, the operation and storage processes in floating-point models hinder the deployment of networks in hardware implements with limited resource and power budgets, such as field-programmable gate arrays (FPGAs) and application-specific integrated circuits (ASICs). To solve this problem, this paper focuses on optimizing the hardware design of CNN with low bit-width integers by quantization. First, a symmetric quantization scheme-based hybrid-type inference method was proposed, which uses the low bit-width integer to replace floating-point precision. Then, a training approach for the quantized network is introduced to reduce accuracy degradation. Finally, a processing engine (PE) with a low bit-width is proposed to optimize the hardware design of FPGA for remote sensing image classification. Besides, a fused-layer PE is also presented for state-of-the-art CNNs equipped with Batch-Normalization and LeakyRelu. The experiments performed on the Moving and Stationary Target Acquisition and Recognition (MSTAR) dataset using a graphics processing unit (GPU) demonstrate that the accuracy of 8-bit quantized model drops by about 1%, which is an acceptable accuracy loss. The accuracy result tested on FPGA is consistent with that of GPU. As for the resource consumptions of FPGA, the Look Up Table (LUT), Flip-flop (FF), Digital Signal Processor (DSP), and Block Random Access Memory (BRAM) are reduced by 46.21%, 43.84%, 45%, and 51%, respectively, compared with that of floating-point implementation.

## 1. Introduction

Object detection and classification in remote sensing images are hot research topics in earth observation applications. With the development of object detection and classification techniques, a lot of convolutional neural network (CNN)-based methods [1,2,3,4,5,6,7] are adopted in real-time processing systems, such as spaceborne and airborne systems. However, considering the huge volume of remote sensing images and the high requirements for storage and computing resources, deploying these successful CNN models in real-time processing systems is a challenging task [8,9]. To accomplish this task, many researchers adopt a high-performance device as the implementation platform for CNNs. Despite the amazing performance of CNN’s implementation, graphics processing units (GPUs) and central processing units (CPUs) are not very suitable for being loaded in remote sensing systems, as the high power consumption far exceeds the constraints. The field-programmable gate arrays (FPGAs) or application-specific integrated circuits (ASICs) spend less power and provide better performance per watt consumption than GPUs. While the on-chip resources of these platforms are limited, designers still prefer to adopt these platforms in remote sensing applications. However, most state-of-the-art CNN models have the characteristics of intensive complexities and dense calculations, which hinder their deployments in these low-power devices. Therefore, facing the constrains of remote sensing real-time processing platforms, we must provide optimization strategies and corresponding hardware designs to make it possible for CNN-based methods to be implemented in real-time processing platforms.

Recently, there have been a lot of studies on reducing the model complexities of CNNs, and several previous works [10,11,12] proved that convolutional and fully connected operations have a high consumption of computing and storage resources. To reduce these requirements, state-of-the-art methods can be roughly divided into two categories: (1) network structure compression; (2) quantization of CNNs. Both category methods are friendly to hardware implementation.

Extensive studies in the first category are dedicated to the exploration of compact network architectures, which exploit efficient computations. Iandola et al. [13] proposed an efficient macro-architecture, called Fire Module, which used 1×1 size to replace most 3×3 size of convolution kernels to reduce the parameters of CNNs. This strategies have been evaluated on AlexNet [14], and the results showed that the parameters are reduced by 50 times. Howard et al. [15] exploited a similar method to simplify the model using Depth-wise Separable Convolution, which had nearly the same accuracy as VGG-16 [16], with a 32× smaller model size and 27× less computing consumption. Zhang et al. [17] proposed Channel Shuffle for Group Convolutions, which generalized the group convolution and depth-wise separable convolution in a novel form. Maintaining a comparable accuracy, ShuffleNet provided 13× speeds up over AlexNet on an ARM-based mobile device. Some other researchers attempted to eliminate weight redundancies within CNNs. Liu et al. [18] proposed Sparse Convolutional Neural Networks (SCNN) to reduce the inherent redundancy. The evaluation result on the PASCAL VOC2007 [19] dataset showed that although SCNN had approximately 2% accuracy degradation, it gained a faster server speed. Denil et al. [20] found that it is feasible to accurately predict up to 95% of weights, with a few determined values for each feature map in several deep learning models. In a similar vein, Wang et al. [21] proposed an improved oriented response network (IORN) using average active rotating filters (A-ARFs). While gaining great performance in oriented prediction, the weights are also compressed 4 to 8 times.

In the second category, researchers are exploring another way, which is to quantize the models, converting the floating-point precision into lower bit-width fixed-point numerical representation. This type of methods aims at increasing the computation efficiency via approximate multiplications and additions. Gupta et al. [22] proved that it was feasible to train models with 16-bit fixed-point representation, while the degradation of classification accuracy was negligible. Gysel et al. [23] proposed an approximation framework in Caffe [24] using dynamic fixed-point (DFP) quantization and argued that DFP had a more stable accuracy than static fixed-point (SFP). Courbariaux et al. [25] applied a similar approach for classification in three distinct formats: floating-point, fixed-point, and DFP. The results showed that DFP seemed well suited to training deep neural networks. Miyashita et al. [26] proposed logarithmic data representation, which enables CNNs to be encoded to 3 bits with negligible loss in classification performance. Zhou et al. [27] presented an efficient method to convert any pre-trained full-precision CNNs models into a hardware-friendly format, constrained to be either a power of two or zero. Using this method [24], floating-point multiplications can be performed using low-precision multiplications or simple shift operations. Some extreme trends are to represent CNNs with one or two bits. Courbariaux et al. [28] proposed Binary Neural Networks (BNN), where the weights are constrained to only two possible values (e.g., −1 or 1). Li et al. [29] proposed Ternary Weight Networks (TWN) on the basis of BNN, where the weights have one more alternative value 0. BNN and TWN had great benefits to power-hungry components of the digital implementation, converting multiply-accumulate operations into simple accumulations. Moreover, BNN and TWN require 32× or 16× less memory storage than the floating-point models, respectively. Rastegari et al. [30] proposed XNOR-Net, in which activations, as well as weights of BNN were binarized. Experiments showed that while the network provided a 58× speed up and enabled complex models to be deployed on CPU in real time, it still had a significant degradation of classification accuracy on ImageNet.

Consequently, the prior category of approaches is more focused on designing compact network architectures to achieve a high computational efficiency, which has great improvements in some baseline architectures. With the inherent redundancy, it is easy to obtain a high compression ratio of over-parameterized architectures. Notably, these approaches are owned by CNN model designers rather than hardware engineers and have the non-essential contributions to hardware optimization. Instead, a more meaningful challenge for hardware design is to quantize the CNNs that have already gained a trade-off between model complexity and accuracy degradation. While many quantization approaches obtain efficiency improvements to custom hardware, there are still some shortcomings. The approaches that only consider weights quantization [31] mainly focus on memory consumption and less on computational efficiency. Lower bit-depth approximation in BNN, TWN, and XNOR-Net is indeed a hardware-friendly quantization scheme. While converting the floating-point multiplications and additions into shift and count operations is efficient, the substantial accuracy degradation is unacceptable for massive models. With a distinct scaling factor for each layer, DFP successfully optimizes the word length into 6-bit; however, it requires strict dynamic ranges to ensure the correctness of the results. Besides, Jacob et al. [32] proposed a generic quantization method, which is evaluated on several benchmarks and achieves a satisfying performance. However, applying this method in hardware design has a high computing resources requirement.

In this paper, a hybrid-type inference method for CNN implementation in FPGA was proposed, which enables a CNN-based remote sensing image classification network to be successfully deployed in FPGA with limited power and resource budgets. This paper’s contributions can be summarized as follows.

- A symmetric quantization scheme-based hybrid-type inference method is proposed for CNNs. In this method, both feature maps and parameters are quantized into a low bit-depth signed integer. Meanwhile, integer/floating mixed calculations are adopted to efficiently obtain the outputs.
- A training approach for quantized layers is proposed, which reproduces the same hybrid-type algorithm to simulate the behavior of inference. Using this approach, the degradation of model accuracy is reduced when applying the proposed inference method.
- Based on our previous work [33], a hardware architecture is designed to apply the hybrid-type inference in FPGA. In this architecture, a processing engine (PE) for quantized convolutional and fully connected layers is presented. Besides, a fused-layer PE is proposed for state-of-the-art CNNs equipped with Batch-Normalization and LeakyRelu.
- The hybrid-type inference method and training approach are evaluated in five distinct bit-widths on GPU. The results on MSTAR [34] show that the 8-bit hybrid-type obtains a trade-off between optimized bit-width and accuracy degradation. The 8-bit quantized model is implemented in FPGA. The results show that this hardware implementation achieves significant improvements in memory and logical resource consumption.

## 2. Hybrid-Type Inference

The hybrid-type inference method for CNNs was inspired by research work [32]. Unlike [32], we adopt the symmetric quantization scheme [35] to reduce the requirement of computing resources. In addition, some layers of CNN, such as Batch-Normalization (BN) [36] and LeakyRelu [37], are computationally insignificant. However, low bit-width presentation for these layers can have a large impact on performance. Thus, we propose an integer/floating-point hybrid-type CNN inference method. In this section, the details of the proposed method will be described.

### 2.1. Symmetric Quantization Scheme

In the proposed inference method, the symmetric quantization scheme is used to convert floating-point matrices into integer matrices for low-precision calculations. This quantification scheme can be regarded as an affine transform between floating-point matrices and integer matrices, which can be defined as:

(1) r=S×q,

where r represents a floating-point matrix, q represents the corresponding quantized matrix, and S is the quantization parameter scaling factor. For *N*-bit quantization, the elements of q are *N*-bit signed integers. The Scaling factor S in Equation (1) is a floating-point constant and is calculated by the following:

(2) $$S=\frac{\max\left(\left|\max\left(r\right)\right|,\left|\min\left(r\right)\right|\right)}{2^{N-1}-1},$$

where max(⋅) and min(⋅) are used to find the maximum and minimum element from the given matrix, respectively. Using Equation (2), the quantized matrix is calculated by the following:

(3) $$q=\mathrm{clamp}\left(\mathrm{round}\left(\frac{r}{S}\right),-2^{N-1}+1,2^{N-1}-1\right),$$

where round(⋅) means rounding to the nearest integer number. To avoid incorrect representation caused by rounding, the clamp(⋅) function is used to limit the quantized elements to a range of $\left[-2^{N-1}+1,2^{N-1}-1\right]$. 

### 2.2. Quantized Convolutional Layer and Fully Connected Layer

In this section, we discuss the way to convert the dense floating-point multiply-accumulate convolutional operations into efficient integer operations under the symmetry quantization scheme. Considering the similarities between the fully connected layer and convolutional layer, we only derive the quantized calculation method for the convolutional layer here. The final conclusion for the fully connected layer is given directly. The convolution is defined as Equation (4) [38], where x and w indicate the input feature maps with a size of *H* × *W* and kernel matrices with a size of *K* × *K*, y indicates the output feature maps of the convolutional layer, and I indicates the number of input channels in x.

(4) $$y_{h,w}^{j}=\sum_{i}^{I}\sum_{kh=0}^{K-1}\sum_{kw=0}^{K-1}x_{h+kh,w+kw}^{i}\times w_{kh,kw}^{ij},$$

The symmetry quantization scheme is applied to weights and input feature maps. After obtaining the maximum and minimum values of all weights, the scaling factor $S_{w}$ of the weight matrices can be calculated by Equation (2). For the input feature maps, it is ineffective to find the maximum and minimum values during the inference phase. Therefore, the maximum and minimum values are estimated during the training phase. The details will be discussed in Section 3.1. With these estimations, the scaling factor $S_{x}$ of the input feature maps can be calculated by Equation (2). According to the above description and Equation (1), Equation (4) can be rewritten as:

(5) $$y_{h,w}^{j}=\sum_{i}^{I}\sum_{kh=0}^{K-1}\sum_{kw=0}^{K-1}S_{x}q_{x_{h+kh,w+kw}^{i}}\times S_{w}q_{w_{kh,kw}^{ij}},$$

where $q_{x}$ and $q_{w}$ denote quantized input feature maps and quantized weights, respectively. The scaling factors $S_{x}$ and $S_{w}$ are the same constants for all elements of the summation; therefore Equation (5) can be rewritten as:

(6) $$y_{h,w}^{j}=S_{x}S_{w}\sum_{i}^{I}\sum_{kh=0}^{K-1}\sum_{kw=0}^{K-1}q_{x_{h+kh,w+kw}^{i}}\times q_{w_{kh,kw}^{ij}},$$

In the *N*-bit quantization, the elements of $q_{x}$ and $q_{w}$ are *N*-bit signed integers. Considering *N*-bit multiplication, the bit-width of these multiplication products is expanded into *2N*-bit. To prevent the accumulation results from overflowing, a 32-bit accumulator is used to collect the multiplication products.

Bias-addition is a general operation in the convolutional layer, and Equation (6) can be added with the quantized bias array to merge it into layer computation directly. This operation, fused in the layer computation, can have benefits in hardware optimization. The bias array is quantized into *N*-bit signed integers using Equation (3), where the scaling factor $S_{b}$ is defined as:

(7) $$S_{b}=S_{x}S_{w},$$

Then, the fused layer is defined as:

(8) $$y_{h,w}^{j}=S_{b}\left(\sum_{i}^{I}\sum_{kh=0}^{K-1}\sum_{kw=0}^{K-1}q_{x_{h+kh,w+kw}^{i}}\times q_{w_{kh,kw}^{ij}}+q_{b^{j}}\right),$$

The products of the accumulator are the quantized output feature maps and Equation (8) shows the de-quantization operation to obtain the floating-point output feature maps.

As for the fully connected layer, the responses are defined as Equation (9) [38], where $x^{i}$ is the ith input neuron, $y^{j}$ is the jth output neuron and I represent the collection of input neurons. Besides, $w^{ij}$ denotes the weight from the ith input neuron to the jth output neuron and $b^{j}$ denotes the bias for the jth output neuron

(9) $$y^{j}=\sum_{i}^{I}x^{i}\times w^{ij}+b^{j},$$

Analogy to the convolutional layer, the quantized calculation method for the fully connected layer is defined as:

(10) $$y^{j}=S_{b}\left(\sum_{i}^{I}q_{x^{i}}\times q_{w^{ij}}+q_{b^{j}}\right),$$

With the proposed quantization scheme, all the parameters of the convolutional layer and fully connected layer, involved in the inference are converted to *N*-bit signed values, which is of great benefit for implementation in FPGA. Compared to the floating-point type, the requirement of parameter storage is reduced to 32/*N*, while the bandwidth requirement of parameters in calculation also becomes 32/*N* of the original. 

### 2.3. Integer/Floating-Point Hybrid-Type Inference

**Figure 1:**
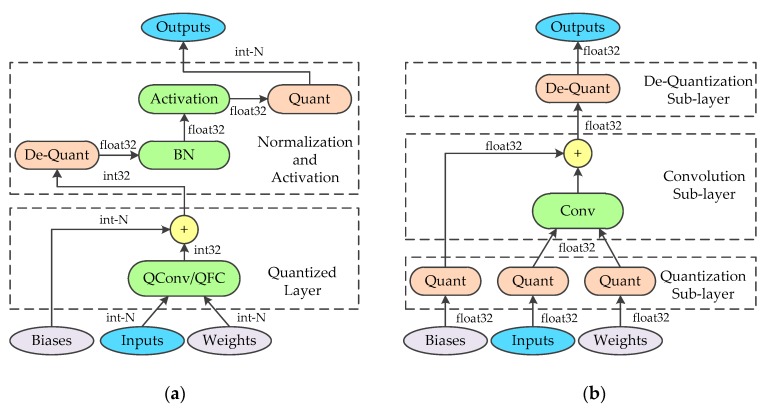
(**a**) Integer/floating-point hybrid-type inference in FPGA. (**b**) The architecture of the quantized convolutional layer during training.

Most of the current state-of-the-art convolutional neural networks adopt BN to accelerate training and LeakyRelu Activation to solve the problem of the vanishing gradient. However, the symmetric quantization scheme is unsuitable for approximate calculation of these functions. BN requires high computational precision and the outputs of LeakyRelu need to be rescaled using quantization. Therefore, in this paper, the integer/floating-point hybrid-type inference method is adopted. In this method, the convolutional and fully connected layers are calculated by using signed integer data type, while the floating-point normalizations and activations are maintained. Figure 1a shows the hybrid-type inference. When applying this method in hardware implements, adjacent identical operations are merged to optimize the hardware design. To be specific, the floating-point multiplications of BN and de-quantization are fused into one operation and perform the same optimization for activation and quantization. This optimization is helpful to reduce the use of the floating-point multiplier. The hardware details will be discussed in Section 4.2.

## 3. Training Approach for Quantized Layers

The most convenient approach to the implementation of the hybrid-type inference in FPGA is to directly quantize the trained floating-point parameters. However, this approach is not recommended, as we found that it leads to serious accuracy drops for some network outputs. Since it is necessary to select the largest boundary values in the same layer for quantization, the relative error will be higher for smaller weights. Moreover, if there is a value that is particularly large or small compared to other weights, the quantization resolution and accuracy of all quantization results will seriously decrease. To solve this problem, a training approach for quantized layers is proposed, which reproduces the same quantization algorithm used in the inference and simulates the behavior of the quantized layers. In this section, we discuss the forward-propagation and backward-propagation algorithms.

### 3.1. Forward-Propagation

In this paper, the symmetric quantization scheme is applied to quantize the convolutional layer and the fully connected layer. Their forward-propagation algorithm is similar. For simplicity, we only discuss the forward-propagation algorithm of the quantized convolutional layer. In the proposed training approach, the convolutional layer is replaced by a custom quantized convolutional layer. As shown in Figure 1b, the custom layer is composed of three sub-layers, where the quantization and de-quantization sub-layer are fused into a generic convolutional layer. The forward-propagation of the quantization sub-layer can be divided into two parts: calculating the scaling factor and obtaining the quantized value. As the weights are uniquely determined in each training step, the scaling factor of weights can be calculated by the boundary values of the parameters using Equation (2). To prevent the product of the infinity, the scaling factor is compared with an epsilon and the result is determined by the maximum value. With the scaling factor, the quantized weights can be calculated using Equation (3). While the quantization of the input features is consistent in principle with the weights, the acquired boundary values are slightly different. If the boundary values are determined for each input image separately, additional image traversal operations are required. While the operation has little influence on training, considering the correspondence between inference and training, the same operation must be employed during the inference. This means that the boundary values of the features are calculated online, which introduces a large amount of calculations. To reduce the calculations during the inference, the boundary values of the input features for each quantized convolutional layer are estimated to obtain the scaling factor of the input features off-line for the inference. Since the estimated boundary values are hardly going to be acquired from the whole training set directly, the mini-batch training strategy and exponential moving averages (EMA) are adopted. To get the estimation of boundary values, the mean of the boundary values in each mini-batch are collected and aggregated via EMA across thousands of training steps. The mean boundary values of the mini-batch are used as the quantization parameters for training, while the estimated values are used for the inference. The forward-propagation algorithm of the quantization sub-layer for input features is described in Algorithm 1, where ε is the epsilon, and λ indicates the momentum of EMA. As for the biases, the scaling factor is calculated according to Equation (7), and the quantization process is the same as the weights. It is necessary to record the scaling factor of the input features and weights, which is applied in the de-quantization sub-layer. Since the convolution sub-layer is a generic convolutional layer with no modification, we do not discuss its forward-propagation stage here. The only calculation of the de-quantization sub-layer is that of multiplying the results of the convolution sub-layer by the scaling factor of the biases.

**Algorithm 1** The forward-propagation algorithm of quantization sub-layer for input features**Input**:Values of input features over a mini-batch: $B=\left\{x_{1},x_{2},\ldots ,x_{m}\right\}$;
Boundary values to be estimated: max_moving,min_moving**Output**:
$\left\{q_{idx}=\mathrm{Quantization}\left(x_{idx}\right)\right\}$

**Step1.**
$max\_x=\frac{1}{b}\sum_{idx=0}^{idx=b-1}\max\left(x_{idx}\right)$,$min\_x=\frac{1}{b}\sum_{idx=0}^{idx=b-1}\min\left(x_{idx}\right)$ //Real Boundary values
**Step2.**
max_moving=(1−λ)⋅max_x+λ⋅max_moving, 
max_moving=(1−λ)⋅max_x+λ⋅max_moving //Moving Boundary values
**Step3.**
$S_{x}=\frac{\max\left(\left|max\_x\right|,\left|min\_x\right|\right)}{2^{N-1}-1}$ //Scaling factor
**Step4.**
$q_{idx}=\frac{x_{idx}}{\max\left(S_{x},\varepsilon \right)}$ //Quantization

### 3.2. Backward-Propagation

The trainable parameters of the convolutional layer are updated as follows [38]:

(11) $$\left\{ \begin{array}{l} w_{l+}^{ij}=w_{l}^{ij}-\lambda \frac{\partial loss}{\partial w_{l}^{ij}}\\ b_{l+}^{ij}=b_{l}^{ij}-\lambda \frac{\partial loss}{\partial b_{l}^{ij}} \end{array}\right.,$$

The common approach to calculating the derivatives of the loss function with respect to the parameters is to use the delta rule [39]. In the rule, the derivatives are acquired using the sensitivity matrices δ of each layer, which are back-propagated from the following layers. Therefore, the sensitivity transmission rules for the quantized layer need to be defined first. The following Equation (12) shows the recurrence relation of the sensitivity matrices for the generic convolutional layer.

(12) $$\delta_{l-1}^{i}=\sum_{j\in M_{j}}w_{l}^{ij}\times \delta_{l}^{j},$$

where $\delta_{l}^{j}$ denotes the jth sensitivity matrix of the lth layer, while $\delta_{l-1}^{i}$ denotes the ith sensitivity matrix of the previous layer. The delta rule for updating weights assigned to a given output feature map is equal to a copy of the inputs, convoluted by the corresponding sensitivity matrix. That is to say,

(13) $$\frac{\partial E}{\partial w_{l}^{ij}}=x_{l-1}^{i}\times \delta_{l}^{j},$$

where E is the total loss. The bias is added to the corresponding output channel. Thus, its contribution to the error is reflected by all of neurons within the same channel. Using the same rule, the bias gradient can be immediately computed by simply summing over the items in $\delta_{l}^{j}$.

(14) $$\frac{\partial E}{\partial b_{l}^{j}}=\sum \delta_{l}^{j},$$

With Equations (11)–(14), we begin to discuss the behavior of the backward stage for the quantized convolutional layers. In essence, quantization operation only converts the data type into the integer, without changing the computation within layers. Deriving the backward-propagation algorithm, only minor changes need to be applied to the original. To write it more easily, QT(⋅) and DQT(⋅) are used to denote the quantization and de-quantization function, respectively. The responses of the quantized convolutional layer can be simplified as

(15) $$x_{l}^{j}=DQT(\sum QT(x_{l-1}^{i})\times QT(w_{l}^{ij})+QT(b_{l}^{j})),$$

To use the delta rule, the recurrence relation of sensitivity needs to be defined for the quantization and de-quantization sub-layers first. For QT, the outputs are calculated by the copy of inputs, scaled by the scaling factor with rounding and clamping in the forward stage. Regardless of the effects of rounding and clamping, the gradient-direct-pass strategy is used for these functions. Thus, the output sensitivity matrices of the quantization sub-layer are simply the input sensitivity matrices, scaled by the same input scaling factor.

(16) $$\delta_{s-1}^{i}=\frac{1}{S_{x}}\cdot \delta_{s}^{i},$$

where $\delta_{s}^{i}$ and $\delta_{s-1}^{i}$ denote the input and output sensitivity matrices of the quantization sub-layer, respectively. Similarly, the output sensitivity matrices of de-quantization sub-layer are defined as:

(17) $$\delta_{s-1}^{i}=S_{x}S_{w}\cdot \delta_{s}^{i},$$

Since the quantization-convolution-de-quantization structure is adopted in the forward stage, the sensitivity matrices are iterated from finish to start. For the middle convolution sub-layer, the only thing to modify is that the weights are replaced by quantized values. The recurrence relation of sensitivity for the whole quantized convolutional layer can be calculated by the following:

(18) $$\delta_{l-1}^{i}=S_{x}S_{w}\cdot \sum_{j\in M_{j}}QT(w_{l}^{ij})\times \delta_{l}^{j}\cdot \frac{1}{S_{x}}=S_{w}\cdot \sum_{j\in M_{j}}QT(w_{l}^{ij})*\delta_{l}^{j},$$

where M represent the collection of input feature maps. The above Equation (18) indicates that the sensitivity matrices for the previous layer are the convolution products of the quantized weights with the input sensitivities, scaled by the scaling factor of weights. The gradients of the weights and biases can be calculated by the following:

(19) $$\{\begin{array}{c} \frac{\partial E}{\partial w_{l}^{ij}}=\frac{\partial E}{\partial DQT(u)}\cdot \frac{\partial DQT(u)}{\partial u}\cdot \frac{\partial u}{\partial QT(w_{l}^{ij})}\cdot \frac{\partial QT(w_{l}^{ij})}{\partial w_{l}^{ij}}\\ \frac{\partial E}{\partial b_{l}^{j}}=\frac{\partial E}{\partial DQT(u)}\cdot \frac{\partial DQT(u)}{\partial u}\cdot \frac{\partial u}{\partial QT(b_{l}^{j})}\cdot \frac{\partial QT(b_{l}^{j})}{\partial b_{l}^{j}} \end{array},$$

where

(20) $$u=\sum QT(x_{l-1}^{i})\times QT(w_{l}^{ij})+QT(b_{l}^{j}),$$

With the gradient-direct-pass strategy, derivatives of the QT and DQT, with respect to their inputs, are equal to their scale factors. Therefore, the derivatives for updating the parameters equal:

(21) $$\left\{ \begin{array}{l} \frac{\partial E}{\partial w_{l}^{ij}}=S_{x}S_{w}\cdot \left(\delta_{l}^{j}\times QT\left(x_{l}^{i}\right)\right)\cdot \frac{1}{S_{w}}=S_{x}\cdot \left(\delta_{l}^{j}*QT\left(x_{l}^{i}\right)\right)\\ \frac{\partial E}{\partial b_{l}^{j}}=S_{x}S_{w}\cdot \sum \delta_{l}^{j}\cdot \frac{1}{S_{x}S_{w}}=\sum \delta_{l}^{j} \end{array}\right.,$$

The recurrence relation of sensitivity for the quantized fully connected layer can be calculated by the following:

(22) $$\delta_{l-1}=S_{w}\cdot {\left(QT(w_{l})\right)}^{T}\cdot \delta_{l},$$

The analogous expressions of the derivatives are:

(23) $$\left\{ \begin{array}{l} \frac{\partial E}{\partial w_{l}}=S_{x}\cdot {\left(QT\left(x_{l-1}\right)\right)}^{T}\cdot \delta_{l}\\ \frac{\partial E}{\partial b_{l}}=\delta_{l} \end{array}\right.,$$

## 4. Implementation of Hybrid-Type Inference

### 4.1. Implement Architecture in FPGA

**Figure 2:**
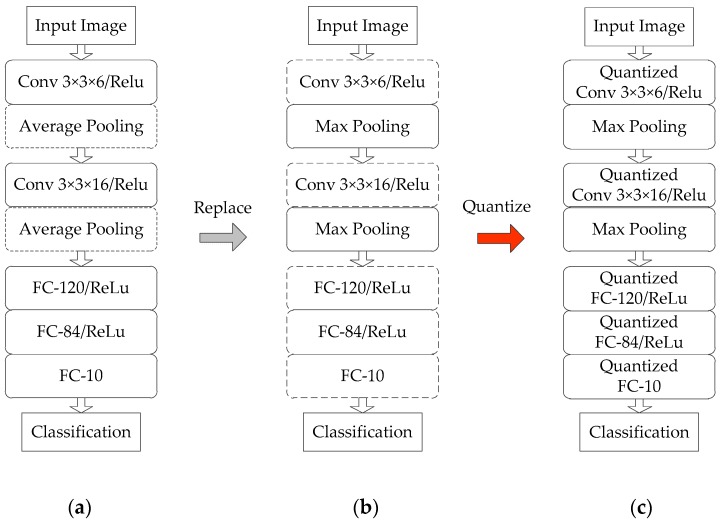
Frameworks of the fundamental network (**a**) modified network (**b**) and quantized network (**c**).

A modified LeNet-5 [38] network framework is adopted in our previous work [33], which performs well for remote sensing images classification. The implementation architecture in [33] has successfully deployed the modified network on FPGA. In this paper, the above-mentioned network framework and implementation architecture is adopted as our benchmarks. The benchmark framework supports ten classifications for input images of a size of 126 × 126. The classification accuracy tested on the MSTAR [34] dataset reaches 98.18%. In this paper, slight modifications are made to the fundamental network. The average pooling is replaced by the max pooling. Meanwhile, the convolutional layer and fully connected layer are quantized. These modifications are helpful to optimize the hardware design. Figure 2 shows architectures of the fundamental network, modified network, and quantized network.

**Figure 3:**
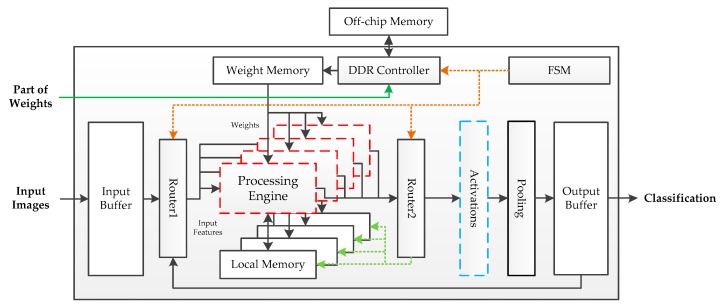
Hardware implementation architecture on the FPGA platform.

The fundamental hardware implementation architecture is shown in Figure 3. The off-chip memory is a Double Data Rate (DDR) SDRAM, which is used to store parameters. Except for the first fully connected layer, the volume of the parameters is tiny. Thus, the weights of the first fully connected layer are put into off-chip storage after power-on, while other parameters are stored in the on-chip Read-Only Memory (ROM). Pipeline processing is adopted in this architecture. Four PEs are used to achieve the calculation, each with a local memory to accumulate the intermediate results. When images are fed into the buffer, the finite state machine (FSM) controls the system to enter working mode. The processing engines get features though router1 and read weights from the weight memory. The output data flow process engines are determined by router2. If the current calculation results are layer responses, these results will be sent into the activation module; otherwise, they will be stored in the local memory for overlap-addition. An output buffer is used to receive the feature maps from the pool module during the inference phase and transmit the final classification prediction at the output stage. While the above architecture performs well in the deployment of the benchmark framework, it employed floating-point operations for implementation, which results in high logical resource and memory consumption.

### 4.2. Hybrid-Type Processing Engine

**Figure 4:**
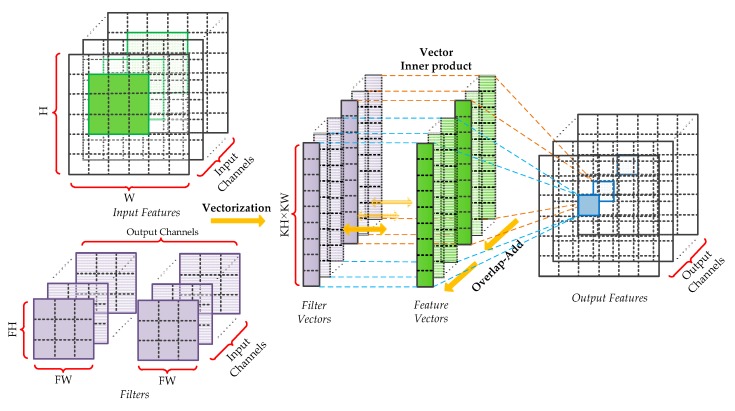
Efficient calculation method for the convolutional and fully connected layers.

**Figure 5:**
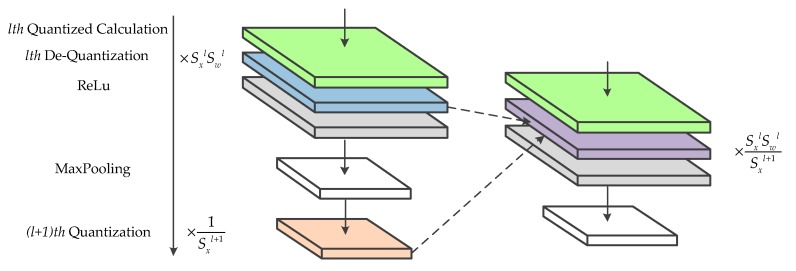
Fusing floating-point multiplication for the optimization to hardware design.

In this section, we focus on designing the PEs for the hybrid-type inference to reduce the logical resource consumption and memory footprint. An efficient calculation method for the convolutional layers and the fully connected layers is introduced in our previous work [33], which is composed of vector inner products and overlap-additions. As shown in Figure 4, the patches of input features and filters are converted to vectors first. Then, the vector inner products, between input vectors and the corresponding filter vectors, are performed to get the intermediate results. Finally, these results are accumulated using the overlap-add method to obtain the output pixel. In this paper, the design of hybrid-type PEs uses the same method. Moreover, as the quantized framework adopts Relu activation and Max pooling without BN, the quantization, and de-quantization operations can be merged to optimize the design of PE. Since the symmetric quantization is used, the sign bit of quantized values is consistent with the corresponding real values and the magnitude relation between pixels has not been modified. Therefore, the latter quantization sub-layer can be put before the activation function and the pooling layer. Considering that both quantization and de-quantization involve floating-point multiplication, the hardware design can be optimized by fusing the same operations. To be specific, the adjacent scaling factors are converted into a new value and calculate the floating-point input feature by only one multiplication, which is shown in Figure 5.

**Figure 6:**
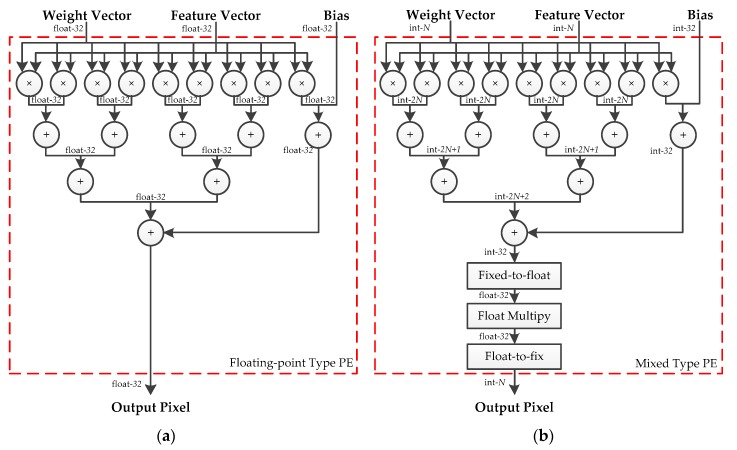
(**a**) Structure of the floating-point-type PE. (**b**) Structure of the hybrid-type PE.

The structures of floating-point-type and hybrid-type PE are depicted in Figure 6. The floating-point addition and multiplication units are replaced by the corresponding fixed-point units without changing the interconnections inside the module. Considering the overflow of the fixed-point addition in the extreme situation, the resulting width of each stage is increased by 1 bit. A notable exception is the output stage. The overlap-add method is used to accumulate convolution calculations from different input channels, and the resulting width of the addition at the output stage is defined as 32-bits to avoid overflow. A fixed-to-float module is used to convert the 32-bit integers into 32-bit floating-point values for the following floating-point multiplication. Meanwhile, a float-to-fixed module is adopted to get the N-bit output feature maps for the following layer. The proposed floating-point-type PE and five distinct bit-width hybrid-type PEs are synthesized, using Xilinx Vivado Design Suite 2017.2, respectively. The results of the logical resource consumptions are listed in Table 1. Compared with the floating-point type, hybrid-type PE has a huge advantage in resource occupation of Look Up Table (LUT), Flip-flop (FF) and Digital Signal Processor (DSP).

**Figure 7:**
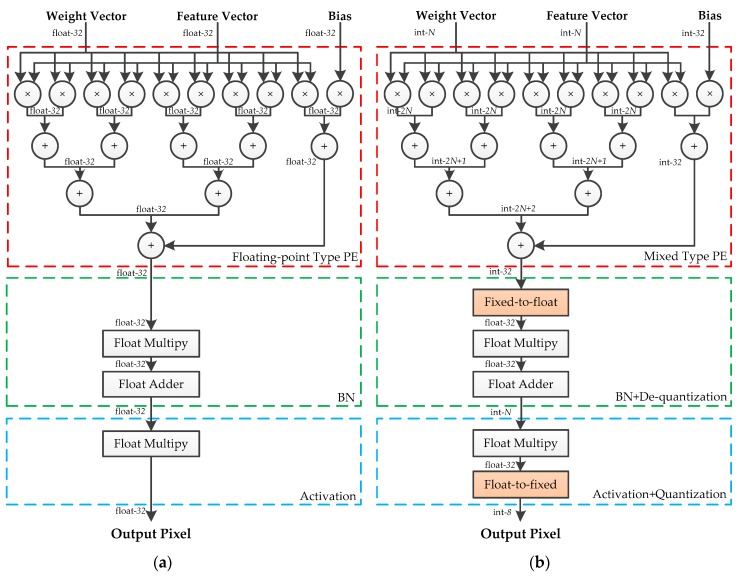
(**a**) Structure of the fused-layer floating-point-type PE. (**b**) Structure of fused-layer hybrid-type PE.

State-of-the-art neural networks are usually equipped with BN and LeakyRelu. BN is used to normalize the responses of the convolutional layer, and LeakyRelu is used to activate the output of the BN. In this case, the convolutional layer, BN, and LeakyRelu activation can be taken into consideration together. A fused-layer PE is designed to apply the proposed method to these state-of-the-art neural networks, which is shown in Figure 7. The outputs of BN are defined as:

(24) $$y=\gamma \frac{x-\mu }{\sigma }+\beta ,$$

where μ and σ denote the mean and standard deviation estimation of the input features. γ and β are the parameters of BN. Both BN and the de-quantization involve multiplications. These multiplications can be merged into one operation to reduce the use of the floating-point multiplier. Using Equation (8), the output of BN can be calculated by the following:

(25) $$y=aq_{x}+b,\mathrm{with}a=\frac{\gamma \cdot S_{b}}{\sigma }\mathrm{and}b=\left(\beta -\frac{\gamma }{\sigma }\mu \right),$$

For a trained model, a and b can calculated off-line. Therefore, only one multiplier, one adder, and a fixed-to-float module are required to achieve the fused operation. Since the multiplication coefficient of LeakyRelu is determined by the outputs of BN, the above-mentioned optimization is unsuitable for combining the calculation of BN and LeakyRelu. However, the quantization of the following layer can be fused into LeakyRelu in the current layer. LeakyRelu activation is defined as:

(26) $$\mathrm{LeakyRelu}(x)=\left\{ \begin{array}{l} x,x\geq 0\\ \alpha x,x<0 \end{array}\right.,$$

Using Equation (3), the fused operation is written as:

(27) $$q_{x}^{l+1}=\left\{ \begin{array}{l} \frac{1}{S_{x}}y^{l},y\geq 0\\ \frac{\alpha }{S_{x}}y^{l},y<0 \end{array}\right.,$$

The products of $1/S_{x}$ and $\alpha /S_{x}$ are calculated off-line and stored in the on-chip memory. When performing this optimization, an additional multiplexer is used to select the corresponding multiplication coefficient. A set of experiments on the fused-layer PEs is conducted, and Table 2 shows the logical resource consumption of fused-layer PEs. Compared with the floating-point PEs, the hybrid-type fused-layer PEs achieve a significant reduction in the logical resource consumption of FF, LUT, and DSP. 

## 5. Experiments and Results

We conduct experimentation in two parts. In the first part, five different bit-width quantized networks are trained on the GPU to obtain the optimal bit-width that is most suitable for hardware implementation. Another part of the experimentations aims to implement the quantized network in FPGA and compare it with the fundamental network described in Section 4 in terms of the on-chip logical and memory resources. The latter indicated that it is easier to deploy the hybrid-type inference in FPGA.

### 5.1. Dataset Description and Data Preprocessing

**Figure 8:**
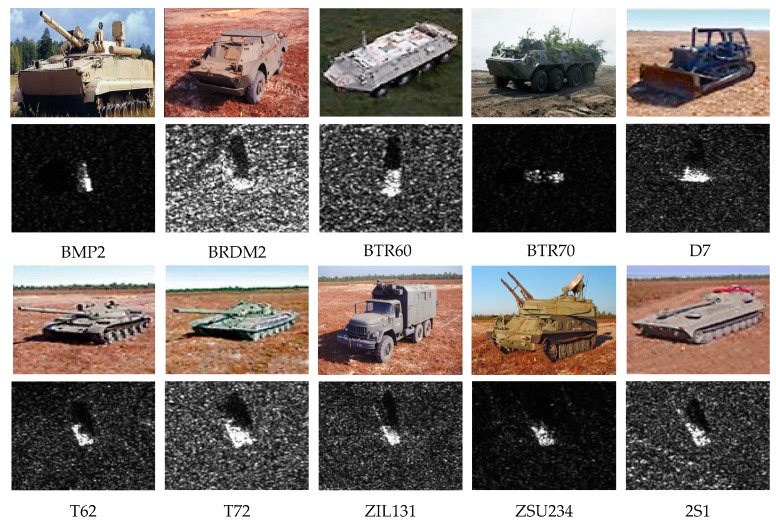
Samples of each type of vehicle in MSTAR with corresponding optical images.

The proposed inference method is evaluated on the MSTAR dataset [34], which was jointly provided by Defense Advanced Research Projects Agency (DARPA) and Air Force Research Laboratory (AFRL). The MSTAR dataset was composed of massive ground military vehicle chips of ten types and collected by an X-band Synthetic Aperture Radar (SAR) in one-foot resolution spotlight mode, with full aspect coverage. All the chips were extracted from the original SAR image and had a fixed size of 128×128. The sample vehicle chip of each type, with the corresponding optical image is depicted in Figure 8. The MSTAR dataset is divided into two parts: 2747 training samples and 2425 testing samples. The statistical result of each part is listed in Table 3. In the fundamental architecture, the image has a fixed size of 126×126, which is determined by the network. Therefore, central cropping is adopted on the images to meet the requirement. As the target is in the center of the images in the MSTAR dataset, the clipping method would not cause a serious loss of information.

### 5.2. Quantized Training with Different Bit-width

The floating-point-type modified network is trained as a baseline. Meanwhile, the bit-widths *N* of quantized networks are set to 4, 6, 8, 10, and 12 for experimentation. All experiments are implemented in the Pytorch 0.4.0 framework with Torchvision 0.2.1. As for the training and testing platform, a work-station with two Intel Xeon E5-2697 v2 CPUs and one NVIDIA TITAN X GPU is used. The optimizer used in this paper is SGD (Stochastic gradient descent) with a learning rate of 0.01 and weight decay of 5×10^−4^. To avoid overfitting, dropout is used for the first and second fully connected layers with a dropout rate of 0.5. For the experiments of quantized networks, the momentum of EMA is 0.9. All weight parameters of the above networks are initialized by random values and trained for 150 epochs, with a batch size of 64. We repeated each experiment five times and took the mean of the results as the final conclusions.

Table 4 shows the classification accuracies of the fundamental model and quantized model in five data formats. The model with the floating-point data type obtains the greatest result of 98.43%. The classification accuracy of the 4-bit quantized model has the maximum loss of 4.0%. The other quantized models obtain satisfying performance and the average results are all around 97.2%, with a 1.2% accuracy degradation, compared to floating-point types. It can be seen that the classification accuracy degradation is reduced as the bit-width increases, which may be due to the increased quantization resolution resulting in a computing error reduction. However, the performance can hardly improve after reaching the maximum as the inherent redundancy within the network is limited. The standards deviations of 4-bit and 6-bit quantized models are higher than the others. This means that the stability of training is not good when adopting a low bit-width quantization scheme and multiple trainings are required to obtain a satisfying performance. The memory requirement for the weight storage of quantized models is shown in Table 5. These results indicate that the compression rate of 4, 6, 8, 10, and 12-bit quantization is 8.0×, 5.3×, 4.0×, 3.2× and 2.7×, respectively. Since the memory requirement of scaling factors is negligible, the compression rate η can be approximately calculated by the following:

(28) $$\eta =\frac{32bit}{Nbit},$$

The image classification performance will degrade when the network is quantized. However fortunately, it is within an acceptable range. More importantly, the requirement of on-chip Static Random-Access Memory (SRAM) and memory bandwidth sharply decreases. The 8-bit quantized model achieves a trade-off between classification accuracy degradation and this requirement; therefore, the 8-bit quantization scheme is used for the hardware optimization design in this paper.

**Figure 9:**
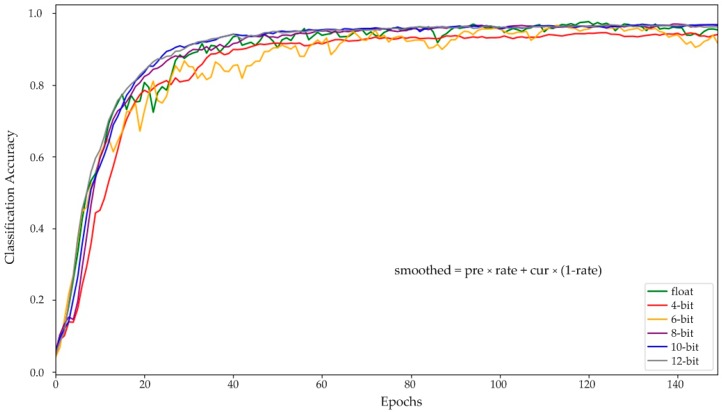
Training processes of the fundamental floating-point-type and quantized-type networks.

Figure 9 shows the test results for each epoch for all the aforementioned models. To clearly reflect the test results, the formula, described in the figure, is used to smooth the curve, and the smoothing rate is set to 0.65. As can be seen from the results, floating-point networks achieve nearly best performance at about 50 epochs, while all the quantized networks require about 130 epochs. This result indicates that quantized CNN models need to be trained for a longer period to achieve the best performance.

A set of experiments is conducted to illustrate that the quantized network has a better performance, using the proposed training approach, than the directly quantized network. Since it is unachievable to directly apply the symmetric quantization scheme, without the scaling factor for each quantized layer, non-updating training is adopted to simulate the direct quantization. There is no change in weights after the non-updating training, as the learning rate is set to zero. Meanwhile, the scaling factor of the input features can be calculated using the estimation of boundary values through training steps. The directly quantized networks are trained for 10 epochs, and the parameters of each model are initialized by the weights of the fundamental floating-point model, which has the best performance, at 98.55%. Table 6 shows that all the directly quantized networks have a serious accuracy degradation of more than 50%, compared to the quantized models, using the proposed training approach. Therefore, deploying the quantized models on hardware platforms has a high demand on the proposed training approach to obtain the best performance.

**Figure 10:**
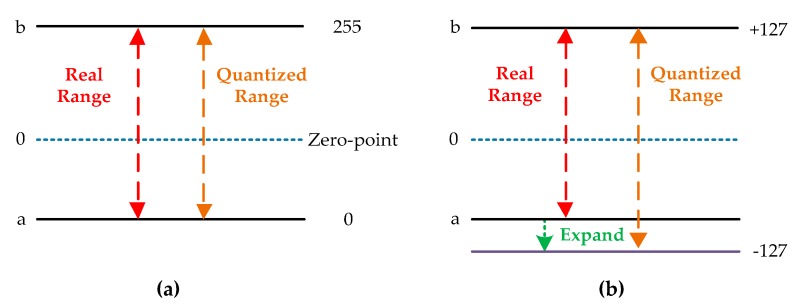
(**a**) Real dynamic range and quantized range in [32].(**b**) Real dynamic range and quantized range in ours.

We conducted another set of experiments to compare the proposed quantization scheme with [32]. The quantization scheme with zero-point and co-design training approach proposed in [32] are reproduced under the Pytorch framework. Five data formats are used to quantize the fundamental network. The training platform and details of the training parameters are the same as in the first set of experiments. The experimental results, tested on the evaluation set of all quantized networks, are listed in Table 7. The scheme in [32] gains better results, which are 1.36%, 1.08%, 0.56%, 0.44% and 1.05% higher than the proposed scheme, at 4, 6, 8, 10, and 12-bit, respectively. This can be interpreted as higher quantization resolution caused by smaller quantized range, which is depicted in Figure 10. Since scheme in [32] has zero-point (8-bit unsigned integer) to represent the real value of 0, it is unnecessary to expand the dynamic range to distinguish the sign bit for both weights and features. With the shrinking range, the real values get a refined representation and computational error is reduced. However, this scheme involves more calculation than ours when performing the optimization of hardware design. The quantization strategy adopted by [32] can be expressed as Equation (29), where Z denotes the zero-point.

(29) r=S×(q−Z),

The matrix multiplication applied in the convolution and fully connected operations can be performed by the following:

(30) $$q_{3}^{ik}=Z_{3}+\frac{S_{1}S_{2}}{S_{3}}\sum_{j=1}^{N}\left(q_{1}^{ij}-Z_{1}\right)\left(q_{2}^{ik}-Z_{k}\right),$$

The proposed scheme and the scheme in [32] are applied to the fundamental network and compared the number of multiplications and additions in each layer. Table 8 shows that the proposed scheme the same volume of multiplications as scheme in [32] while the number of additions is reduced by 2.87×. This means that adopting the proposed scheme for hardware design would use fewer adders.

### 5.3. Performing Symmetry Quantization in FPGA

In this paper, the 8-bit quantized model and the modified floating-point model with the best performance are deployed for experiments in FPGA. The Xilinx KC705 Evaluation Kit with Xilinx xc7k325tffg900-2 FPGA is used as the implementation platform. The on-board memory is a DDR3 SDRAM with a 64-bit data width and working frequency of 1600MHz. For the quantized model, all the parameters are pre-quantized off-line using the scaling factor calculated through the training phase. Some modification are made to the hardware architecture in [33] when deploying the floating-point model. The pooling module is replaced by a max pooling module, and the full-connected module is reused to reduce the resource consumption. Regarding the quantized model, the floating-point-type PEs are converted into the quantized PEs.

Table 9 shows the results of the above models in relation to the FPGA platform. The modification of network gives an increment of 0.58% on performance and the hardware resource consumptions of LUT, FF and Block Random Access Memory (BRAM) are reduced to 36725, 37,283 and 150, respectively. The classification accuracy of the quantized model tested in FPGA is consistent with the results of tested on GPU, which reflects that our training approach can effectively simulate the behavior of the hardware without any additional accuracy loss. While the 8-bit quantized model has an accuracy degradation of 0.99%, compared with the floating-point network, the hardware resource consumptions of LUT, FF, DSP, BRAM are reduced by 46.21%, 43.84%, 45.00% and 51%, respectively. Simultaneously, the proposed quantization scheme gives a decrement of 74.99% on the requirement of the DDR bandwidth. Two implements in this paper have the same processing time of 2.29 ms as that in [33], which means that the hardware design meets the requirements of on-board real-time processing for object classification in remote sensing images. Therefore, implementing CNNs using our method in FPGA helps to get rid of the constraints of limited logical resources and memory bandwidth.

## 6. Conclusions

In this paper, a hybrid-type inference method based on the symmetric quantization scheme is proposed for CNN-based remote sensing image classification. Both feature maps and weight parameters are quantized into low bit-width signed integers in this method. With the signed integer format, CNNs can be efficiently implemented on low-power remote sensing hardware platforms, such as FPGA and ASIC, using low-precision calculations. A training approach for quantized layers is co-designed, which reproduces the same hybrid-type algorithm used during the inference phase to simulate the behavior of quantized calculations to preserve the model accuracy. Finally, the PEs and the fused-layer PEs are designed to implement the proposed method in FPGA. The proposed inference method and training approach are evaluated on Nvidia Titan Xp accelerators. The results tested on the MSTAR dataset shows that the 8-bit hybrid-type gains a trade-off between the optimized bit-width and accuracy degradation. The hybrid-type model and floating-point model are implemented on Xilinx xc7k325tffg900-2 FPGA, and the experimental results show that compared with the floating-point model, the resource consumptions of LUT, FF, DSP, and BRAM are reduced by 46.21%, 43.84%, 45.00% and 51% respectively.

## Figures and Tables

**Table 1:** Logical resource consumptions of PEs in six formats.

Logical Resource	Float	4-bit	6-bit	8-bit	10-bit	12-bit
LUT	2936	396	404	410	417	424
FF	2267	767	824	867	916	961
DSP	38	20	20	20	20	20

**Table 2:** Logical resource consumptions of fused-layer PEs in six formats.

Logical Resource	Float	4-bit	6-bit	8-bit	10-bit	12-bit
LUT	3319	828	836	842	849	856
FF	5517	1512	1569	1612	1661	1706
DSP	42	24	24	24	24	24

**Table 3:** The quantity of training and testing images.

Class	BMP-2	BRDM-2	BTR-60	BTR-70	D7	T-62	T-72	ZIL-131	ZSU-234	2S1
Training	233	298	256	233	299	299	232	299	299	299
Testing	195	274	195	196	274	273	196	274	274	274

**Table 4:** Classification accuracies of floating-point model and quantized models.

No.	Float [33]	4-bit	6-bit	8-bit	10-bit	12-bit
1	98.14%	94.51%	97.07%	97.64%	97.31%	97.15%
2	98.22%	93.97%	96.08%	97.77%	97.15%	97.23%
3	98.55%	95.09%	97.56%	97.19%	97.23%	97.11%
4	98.47%	93.89%	96.82%	97.31%	97.48%	97.03%
5	98.76%	94.68%	97.64%	97.19%	97.36%	97.11%
Mean	98.43%	94.43%	97.03%	97.42%	97.31%	97.13%
Std. dev.	0.22%	0.49%	0.57%	0.24%	0.11%	0.06%

**Table 5:** Weights storage and compression statistics in experiments of quantized models.

	Float	4-bit	6-bit	8-bit	10-bit	12-bit
Weights Storage (MB)	6.638	0.830	1.245	1.660	2.074	2.489
Compression Rate	-	8.0×	5.3×	4.0×	3.2×	2.7×

**Table 6:** Accuracies of directly quantized models and quantized models with training.

	4-bit	6-bit	8-bit	10-bit	12-bit
Quantized with training	94.43%	97.03%	97.42%	97.31%	97.13%
Quantized directly	29.27%	20.70%	42.22%	43.34%	41.17%
Difference	65.16%	76.33%	55.20%	53.97%	55.96%

**Table 7:** Classification accuracies under the proposed scheme and the scheme in [32].

	4-bit	6-bit	8-bit	10-bit	12-bit
[32]	95.79%	98.11%	97.98%	97.75%	98.18%
Ours	94.43%	97.03%	97.42%	97.31%	97.13%

**Table 8:** Number of multiplications and additions in the proposed scheme and the scheme in [32].

Layer	[32]		Ours	
	Multiplication	Addition	Multiplication	Addition
Conv1	922560	2583168	922560	830304
Conv2	921600	1900800	921600	806400
FC1	1728120	5184120	1728120	1728000
FC2	10164	30324	10164	10080
FC3	850	2530	850	840
Total	3583294	9700942	3583294	3375624

**Table 9:** Experimental results of the floating-point model and the 8-bit quantized model in FPGA.

	Available	[33]	Ours	Ours
Format	-	32-bit float	32-bit float	8-bit fixed
Frequency	-	100 MHz	100 MHz	100 MHz
LUT	203800	55745(27.35%)	36725(18.02%)	19753 (9.69%)
FF	407600	45561(11.18%)	37283(9.19%)	20938 (5.14%)
DSP	840	-	220(26.19%)	121 (14.40%)
BRAM (36 Kb)	445	150.5(33.82%)	150(33.71%)	73.5 (16.52%)
DDR Bandwidth	100 Gbps	-	47.62 Gbps	11.91 Gbps
Processing Time	-	2.29 ms	2.29 ms	2.29 ms
Classification Accuracy	-	98.18%	98.76%	97.77%
